# Microbiology education: a significant path to sustainably improve the human and biosphere condition

**DOI:** 10.1093/femsml/uqad013

**Published:** 2023-03-10

**Authors:** Kenneth Timmis

**Affiliations:** Institute of Microbiology, Technical University of Braunschweig, D-38106 Braunschweig, Germany

## Abstract

In this short piece, I connect the dots between the pervasive influence of microbial activities on our health and that of the planet, including their positive and negative roles in current polycrises, our ability to influence microbes to promote their positive influences and mitigate their negative impacts, the roles of everyone as stewards and stakeholders in personal, family, community, national, and global wellbeing, the need for stewards and stakeholders to possess relevant information in order to fulfil their roles and obligations, and the compelling case for microbiology literacy and introduction of a societally relevant microbiology curriculum in school.

## We are all stewards and stakeholders

We are all stewards: of ourselves, our relationships with family and friends and what we do, our communities, nations, the planet (Timmis 2023a). We all know that when we elect to have a hamburger or steak for dinner, it comes with a carbon footprint that has an impact on global warming and climate change, and an environmental footprint that includes eutrophication and oxygen minimum zones, loss of natural habitats and the biodiversity they house (Ceballos et al. 2015), pollution of the environment with pesticides and other biologically active substances, and a number of other consequences. Other daily decisions, such as buying locally-produced goods, have positive impacts. Such considerations matter and constitute the rich tapestry of decisions and actions we make and take throughout our lives that determine how we fulfil our stewardship roles.

And when we personally may not be in a position to take decisions on, for example, national energy policy, subsidies for re-wilding of agricultural land, etc., we are all stakeholders in such policies and have a responsibility to use what civil mechanisms are available to us to both influence such decisions and hold decision-takers to account.

Exerting our stewardship and stakeholder responsibilities is currently more important than ever before because the world is currently faced with multiple existential crises, including global warming and exceeded planetary boundaries (Rockström et al. 2009, Wang-Erlandsson et al. 2022) and tipping points (Timmis 2023a, Lenton et al. 2008, Armstrong McKay et al. 2022, Box et al. 2022; https://www.carbonbrief.org/explainer-nine-tipping-points-that-could-be-triggered-by-climate-change/), crises that exacerbate existing deficits and asymmetries in basic human resources, promote migrations, and can be sources of regional conflicts (Anand et al 2023). There is an urgent need to increase positive impacts and reduce negative impacts in order to lower the probability of humanity destroying the biosphere (Timmis and Hallsworth 2022). But for stewards and stakeholders to effectively fulfil their responsibilities, they need relevant knowledge and adequate understanding of the key underlying issues.

## Knowledge of societally-relevant microbiology is essential to stewards and stakeholders

Microbes and their activities touch us personally in so many different ways, play vital roles in our wellbeing and constitute the life support system of essentially all organisms and indeed of the planet (Timmis et al. 2019). They lie at the centre of the bioeconomy, especially of the discovery and development of new medicines and vaccines, of agricultural yields and food security, of the health of the environment, biodiversity and sustainability (Timmis et al. 2017), and are key actors in the polycrises currently challenging humanity (Cavicchioli et al. 2019, Timmis and Ramos 2021, Timmis et al. 2022; Timmis 2023a). Without understanding the roles of microbes in underpinning important processes that affect us directly and indirectly, we are unable to comprehend what is going on and why, and what are the causes of and potential solutions to needs and problems, and thus to arrive at informed opinions and take optimal decisions. The scale and pervasiveness of microbial influences on decision-relevant processes is huge.

In order to promote their beneficial activities and counteract their negative activities at the personal, family, community, national and global levels, society needs to become literate in societally-relevant aspects of microbiology (Timmis et al. 2019). And the *generational contract* compels us to ensure that the next generation, which will inherit all current problems not solved by us, and all the new ones we create, is fitter than we were to solve the problems they face (Timmis, 2023).

## The International Microbiology Literacy Initiative

The International Microbiology Literacy Initiative (IMiLI) is creating the teaching resources that will constitute an international school curriculum in societally-relevant microbiology (Timmis 2023b). The core of the resources are the class lessons, 300+ *Topic Frameworks* (TFs)—*generic* knowledge frameworks of societally-relevant microbiology topics—organized in 20 Sections: Our Plants, Our Animals, Our Food, Ourselves, Our Health, Our Infections, Our Planet, Global Warming, Our Water, Global Microbiology, Adventures and Discovery, New Frontiers, Microbial Gifts (biotechnology), Their and Our Future, Their and our Past, Our Civilisation and Culture, Our Microbial Friends, Microbial Wellbeing, How We Study Microbes, and Why We Need to be Microbiology Literate.

Unusually for school curricula, the TFs are not only *generic*—each one provides information from which teachers can select and adapt to multiple age groups and teaching aims—but they are also essentially *stand-alone*: understanding the material presented in one does not depend upon material presented in another. As a consequence, teachers can either follow the sequence of TFs as presented in the Sections, or create their own syllabuses by selecting which topics they consider to be the most interesting and suited for their particular teaching aims, and by presenting them in whichever sequence they feel to be most appropriate. This will constitute a significant dimension of empowerment of both teachers, who are stewards of our children's education and development, and students, who are key stakeholders in this process (Timmis 2023b).

TFs will be complemented by TF-specific *Class Experiments*, to enable hands-on immersion of children in the topics taught, recommendations for *Class Excursions* to nearby locations where real-world microbial activities can be experienced close-up (McGenity et al. 2020), *Galleries of Microbial Portraits* that showcase famous and/or important microbes and endow them with personalities (for an example of these portraits, see item 10 of the October Newsletter of the Spanish Society of Microbiology: https://www.semicrobiologia.org/revista-noticiasem/octubre-2022), and other resources.

## The three ‘Rs’: *Relevance, Relevance and Relevance*

As emphasized above, microbial activities are relevant to so many aspects of life. However, microbes are invisible and their activities are mostly under our radar screens, so the importance of microbes is not obvious for most people. Thus, a major aim of the IMiLI curriculum is to reveal the relevance of microbes and awaken a curiosity to discover more: *to spark the flash of insight*. The IMiLI does this by describing the impact of microbes at various levels—the personal level (e.g. for human health, food, culture and cultural diversity), the health of plants and animals, soil and water, for planetary processes, for biodiversity, and so forth.

## Relevance exemplified by two more ‘Rs’: *Romance and Reproduction*

An example of relevance emphasis is a discussion of *Romance and Reproduction—*activities that occupy most people for considerable parts of their lives from the time of puberty (and without which, of course, we would go extinct)—consisting of: *attraction* (gut microbes promote social interactions); *initial impressions* (microbial involvement in olfactory volatile production in the buccal cavity, axillae, feet), *the first meeting* (let's go for a coffee: coffee bean fermentation), *the first date* (a favourite pizzeria: yeast production of dough, cheese, salami, etc.), *first kiss* (the beginning of microbiota exchanges, possibly including Herpes), *the first present* (a box of chocolates: chocolate fermentation), *introduction to friends in a bar* (alcohol, olives fermentation), *introduction to the parents* (flowers: the plant microbiome and health, fertilisers—eutrophication, oxygen minimum zones, biodiversity loss), *deepening intimacy* (more comprehensive exchanges of microbiota; potential for transmission of STIs, including HIV, Monkeypox), *but earlier vaccinations have helped* (HPV vaccine), *progression from romance to reproduction* (the microbiology of reproductive tracts and fertility), *pregnancy* (the mother microbiome and influence of antibiotics), *birth* (vertical transfer of the microbiome), *role of the infant microbiome on immune system development* (breast feeding; family pets; crawling on the floor; cleanliness of the home), and so forth.

This is just one example of how we reveal how microbes are intimately involved, positively and negatively, in our lives in diverse ways at different levels, and how knowledge of these activities can aid us both to understand better what is going on and how we might influence them.

## The IMiLI: a huge international *pro bono* enterprise with ambition

The IMiLI is an international effort of hundreds of microbiologists, undoubtedly one of the largest collective enterprise of microbiologists ever, aiming at nothing less than societally-relevant microbiology being taught in every school in every nation willing and able to embrace it.

## The IMiLI: giving back to society

Most academics, and microbiologists in particular, are an exceptionally privileged breed: they have the pleasure and satisfaction of academic freedom to pursue research directions they consider to be of greatest interest and importance in some of the most beautiful and intellectually stimulating environments, which includes both their institutions and their study sites. The privileged academic life is funded mostly by tax revenues awarded to universities, research institutions and grant agencies, a strategic commitment by society made in the knowledge that educating the young is key to the advancement of human wellbeing, and that research discoveries drive societal development and economic security, providing a good return on investment. This is the *Academic: Society Contract: money in, education of the next generation and discoveries leading to societal benefits out*. The IMiLI, as a thus-far entirely *pro bono* initiative, not only reflects the firm conviction of the hundreds of contributors of the importance of global literacy in societally-relevant microbiology, but also their desire and determination to *give back to society*.

## Relevance to science communication, outreach, lifelong learning and university-level microbiology education

The microbiology teaching resources being created by the IMiLI will not only serve as school curricula, but will also, with minor modification, serve for lifelong teaching and for informal web-based self-teaching. They will also constitute a rich source of off-the-shelf resources for science communication activities of academics and for university: school outreach activities (Gilmore 2021). Indeed, the approach to microbiology teaching adopted by the IMiLI, which emphasizes personal and societal relevance, impacts on sustainability, and implications for stewardship and stakeholder responsibility, may well influence the way some academics teach microbiology in university in future.

## A glimpse in the rear mirror; the seed of the IMiLI was planted over 50 years ago

In 1967, two freshly minted graduates of the University of Bristol who were just starting PhD projects in microbiology decided to offer a weekly course of societally relevant microbiology to the general public within the 1968 programme of the Department of Extramural Studies. The course, entitled *Microbes and Man*, was taught by Martin Collins and Kenneth Timmis and was well attended and received with considerable enthusiasm by Bristolians.

This encouraged us to focus on amplifiers—teachers—and so, the following year, we offered a course entitled *Microbiology for Schools*. This was also well attended by enthusiastic local teachers. At the end of the course, we imagined we had done our job, that microbiology would become part of the school curriculum, and we could concentrate in the final year of our projects on getting results that would merit PhDs being conferred. But of course this was somewhat naïve.

Little did I realise then that, after more than 50 years of a super-exciting academic career in microbiology, I would return to the issue of microbiology in schools/microbiology literacy in society, and re-discover the joy of exploring ways and means of revealing to young people the incredible and exciting world of the microbes and its vital importance to us all.
